# Non-immunosuppressive triazole-based small molecule induces anticancer activity against human hormone-refractory prostate cancers: the role in inhibition of PI3K/AKT/mTOR and c-Myc signaling pathways

**DOI:** 10.18632/oncotarget.12765

**Published:** 2016-10-19

**Authors:** Wohn-Jenn Leu, Sharada Prasanna Swain, She-Hung Chan, Jui-Ling Hsu, Shih-Ping Liu, Mei-Ling Chan, Chia-Chun Yu, Lih-Ching Hsu, Yen-Lin Chou, Wei-Ling Chang, Duen-Ren Hou, Jih-Hwa Guh

**Affiliations:** ^1^ School of Pharmacy, National Taiwan University, Taipei, Taiwan; ^2^ Department of Chemistry, National Central University, Jhong-li, Taoyuan, Taiwan; ^3^ Department of Urology, National Taiwan University Hospital, Taipei, Taiwan

**Keywords:** triazole-base, non-immunosuppression, PI3K/Akt/mTOR signaling, Myc, autophagy

## Abstract

A series of triazole-based small molecules that mimic FTY720-mediated anticancer activity but minimize its immunosuppressive effect have been produced. SPS-7 is the most effective derivative displaying higher activity than FTY720 in anti-proliferation against human hormone-refractory prostate cancer (HRPC). It induced G1 arrest of cell cycle and subsequent apoptosis in thymidine block-mediated synchronization model. The data were supported by a decrease of cyclin D1 expression, a dramatic increase of p21 expression and an associated decrease in RB phosphorylation. c-Myc overexpression replenished protein levels of cyclin D1 indicating that c-Myc was responsible for cell cycle regulation. PI3K/Akt/mTOR signaling pathways through p70S6K- and 4EBP1-mediated translational regulation are critical to cell proliferation and survival. SPS-7 significantly inhibited this translational pathway. Overexpression of Myr-Akt (constitutively active Akt) completely abolished SPS-7-induced inhibitory effect on mTOR/p70S6K/4EBP1 signaling and c-Myc protein expression, suggesting that PI3K/Akt serves as a key upstream regulator. SPS-7 also demonstrated substantial anti-tumor efficacy in an *in vivo* xenograft study using PC-3 mouse model. Notably, FTY720 but not SPS-7 induced a significant immunosuppressive effect as evidenced by depletion of marginal zone B cells, down-regulation of sphingosine-1-phosphate receptors and a decrease in peripheral blood lymphocytes. In conclusion, the data suggest that SPS-7 is not an immunosuppressant while induces anticancer effect against HRPC through inhibition of Akt/mTOR/p70S6K pathwaysthat down-regulate protein levels of both c-Myc and cyclin D1, leading to G1 arrest of cell cycle and subsequent apoptosis. The data also indicate the potential of SPS-7 since PI3K/Akt signalingis responsive for the genomic alterations in prostate cancer.

## INTRODUCTION

Carcinoma of the prostate is one of the most frequently diagnosed cancers and leading causes of cancer death in men. Hormone-refractory prostate cancer (HRPC) is a type of progressive prostate cancer unresponsive to hormone therapy and is one of the major hurdles in clinical oncology although several cancer chemotherapeutic drugs have been developed for clinical treatment [1, 2]. Maximizing treatment options, in particular in long-term surviving patients with decreased disease burden, is of importance. Four pathways are well identified to be responsive for the genomic alterations in prostate cancer that challenges the conversion of localized to androgen-independent metastatic cancers, including phosphoinositide 3-kinase (PI3K) signaling, androgen receptor pathway, rearrangements placing E26 transformation-specific transcription factor family through androgen responsive promoter transmembrane protease serine 2(TMPRSS2) and loss of function of prostate-specific tumor suppressor NKX3.1 [2]. PI3K/Akt is the most extensively investigated pathway in prostate cancer since its involvement is not only clearly identified in localized prostate cancers but also is increased in HRPCs [3, 4]. PI3K/Akt and associated mTOR pathways are responsible for cell survival, growth, metastasis and both chemo- and radio-resistances in prostate cancer and other cancers. Targeting this pathway by inhibitors to increase both chemo- and radio-sensitivities may have great potential in clinical benefits [4, 5].

Sphingosine kinase (SK), a conserved lipid kinase, catalyzes the ATP-dependent formation of sphingosine-1-phosphate (S1P) from the precursor sphingosine [6, 7]. SK1 and SK2 are two major forms found in the cytosol and localized to the nucleus, respectively. S1P may serve as an inducer to regulate diverse cellular functions of cell growth and survival through binding to S1P receptors. It also functions as an intracellular second messenger and a key mediator in cytokine network [6–8]. Recently, targeting the SK/S1P/S1P receptor signaling pathway has been considered as a potential anticancer therapeutic strategy [9]. FTY720, a structural analogue of sphingosine and S1P receptor agonist approved by FDA, is developed as a first line oraldrug of multiple sclerosis through immune-modulating effect [10]. In recent decade, a considerable number of studies have been conducted on FTY720-induced anticancer effect through diverse pathways, including inhibition of histone deacetylase [11], activation of protein phosphatase 2A [12], inhibition of STAT3 signaling [13], up-regulation of death receptor mediating signaling [14] and other pathways [15–18]. Although FTY720 displays immunosuppressive effects through activating S1P receptors, phosphorylation of FTY720 is not necessary to its anticancer activity in numerous cases suggesting the participation of S1P receptor-independent pathways [19]. However, several adverse effects occur to FTY720 treatment [20, 21]. Cardiovascular effects are one of the concerns because of the presence of S1P receptors in the sinus and atrioventricular nodes, myocardial cells, endothelial cells and arterial smooth muscle cells [20]. Therefore, the preservation of anticancer activity but decreasing S1P receptor-dependent immunosuppression is challenging in developing FTY720 analogues.

The introduction of phenylene moiety in a proper position of FTY720 is highly critical for its potent immunosuppressive activity [22]. Therefore, several compounds have been produced after displacing the phenylene ring with other ring moiety and the modification of side chain length (Table 1). SPS-7 (1,3-Dihydroxy-2-((1-octadecyl-1*H*-1,2,3-triazol-4-yl)methyl)propan-2-aminium chloride) is the most effective derivative and the synthetic scheme has been demonstrated (supplementary Scheme I). The signaling of SPS-7 on PI3K/Akt/mTOR/c-Myc pathways and the immune-modulating activitieshave been studied to demonstrate its anticancer potential against HRPC.

**Table 1:** Selected examples for the structure-activity-relationship of 1,2,3-triazole compounds

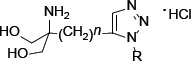				
	*n*	R	PC-3 (IC_50_, μM)	DU-145 (IC_50_, μM)
SPS-1	1	C_6_H_13_	>10	>10
SPS-2	1	C_8_H_17_	>10	>10
SPS-3	1	C_10_H_21_	>10	9.7
SPS-4	1	C_12_H_25_	>10	>10
SPS-5	1	C_14_H_29_	4.9	4.7
SPS-6	1	C_16_H_33_	3.3	5.3
SPS-7	1	C_18_H_37_	3.0	4.6
SPS-8	1	C_20_H_41_	3.8	6.2
SPS-9	2	C_16_H_33_	6.2	nd
SPS-10	2	C_14_H_29_	>10	nd
SPS-11	3	C_14_H_29_	4.2	5.4
SPS-12	4	C_13_H_27_	9.0	6.6
SPS-13	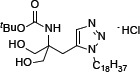		>10	>10
SPS-14	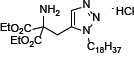		7.9	> 10
SPS-15	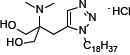		> 10	> 10
SPS-16	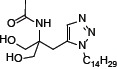		> 10	> 10
SPS-17	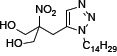		> 10	> 10

Cells were incubated in the absence or presence of the compound 48 hours. The cell proliferation was determined using sulforhodamine B assay.

## RESULTS

### SPS-7 inhibits cell proliferation and induces apoptosis in HRPC cells

**Figure 1:**
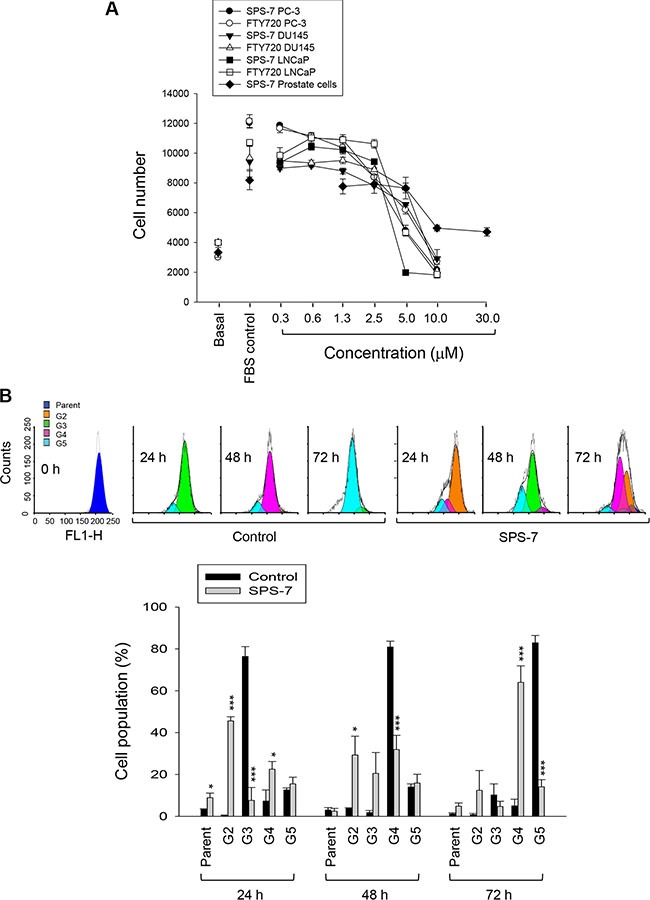
SPS-7 inhibits cell proliferation in human prostate cancer cells (**A**) Cells were incubated in the absence or presence of SPS-7 for 48 h in PC-3 cells and 72 h in DU145, LNCaP and primary prostate cells. After the treatment, cell proliferation was determined using sulforhodamine B assay. Basal, seeding cell numbers. (**B**) PC-3 cells were incubated in the absence or presence of SPS-7 (10 μM) for the indicated time. After treatment, the cells were harvested for flow cytometric analysis of CFSE staining. The cell proliferation was to be followed by monitoring decrease in label intensity in successive daughter cell generations. The proliferation index and the cell populations of parent or different generations were calculated by Modfit LT Version 3.2 and WinList Version 5.0 software. Quantitative data are expressed as mean ± SEM of three to four independent experiments. *P < 0.05 and ***P < 0.001 compared with the control.

Sulforhodamine B colorimetric assay was used to examine the effect on cell proliferation. Both SPS-7 and FTY-720 inhibited cell proliferation with IC_50_ values of 2.98 ± 0.12 *v.s.* 3.26±0.37 μM in PC-3 cells, 4.63 ± 0.15 *v.s.* 6.10 ± 0.55 μM in DU-145 cells, and 2.61 ± 0.10 *v.s.* 3.29 ± 0.12 μM in LNCaP cells, respectively (Figure 1A). The data also showed that the concentration at 10 μM caused a total growth inhibition (cytotoxicity) in these cancer cells. In contrast, the anti-proliferative IC_50_ of SPS-7 in primary prostate cells was 7.0 ± 0.12 μM. The concentrations that caused a total growth inhibition were much higher than 30 μM (Figure 1A). The data indicated the anticancer selectivity of SPS-7. Cell proliferation was further examined in PC-3 cells by CFSE staining, a cell-tracking dye which conjugated to intracellular proteins and was evenly inherited by divided cells after cell proliferation. As a result, the fluorescence-staining was distributed to later generations of the cells with the passage of time (Figure 1B). SPS-7 significantly inhibited cell proliferation, resulting in a profound increase of cell population in earlier generations (Figure 1B). Furthermore, the proliferation index based on the CFSE staining assay was determined showing that the control indexes at 48 and 72 h were 5.7 ± 0.2 and 9.7 ± 0.5, respectively. SPS-7 significantly slowed down the cell proliferation with the indexes of 4.2 ± 0.2 and 4.7 ± 1.0, respectively (*P* < 0.001, *n* = 3).

### SPS-7 induces G1 arrest of the cell cycle and controls the expression dynamics of cell cycle regulator proteins

**Figure 2:**
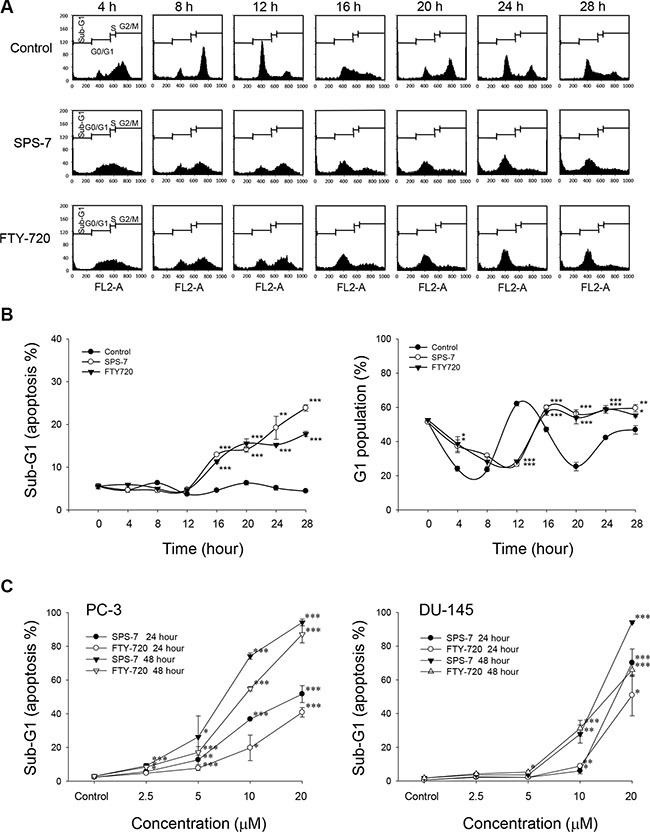
SPS-7 induces G1 arrest of the cell cycle and apoptosis (**A**) Synchronization of PC-3 cells was performed by thymidine block as described in the Materials and Methods section. Then, the cells were released in the absence or presence of 10 μM SPS-7 or FTY720 for the indicated times. Data are representative of three independent experiments. (**B**) Quantitative data are expressed as mean ± SEM of three independent experiments. (**C**) PC-3 or DU-145 cells were incubated in the absence or presence of the compound (10 μM) for 24 or 48 hours. After the treatment, the cells were harvested for the determination of sub-G1 population using the detection of DNA content analyzed with FACScan and CellQuest software. Data are expressed as mean ± SEM of three independent determinations. *P < 0.05, **P < 0.01 and ***P < 0.001 compared with the respective control.

Thymidine overload in thymidine block assayhalted the DNA replication and synchronized PC-3 cells mainly at late G1 and S phases. After the release from thymidine block, the cells proceeded through the stages of cell cycle with the majority at G2/M phase and G1 phase after the release for 8 h and 12 h, respectively (Figure 2A). Both SPS-7 and FTY-720 retarded the progression of cell cycle and, once the cells progressed into G1 phase, the cell cycle was arrested and the population at sub-G1 phase (apoptosis) was subsequently increased (Figure 2A). The quantitative data also demonstrated the sustained high levels of G1 phase population and the increases of apoptosis under the exposure to SPS-7 and FTY-720 (Figure 2B). Similar effects on G1 arrest were obtained in DU-145 cells (control of 46.8 ± 2.2% compared with 60.2 ± 1.2% and 57.6 ± 0.9% for SPS-7 and FTY-720, respectively; *P* < 0.001, *n* = 3). The concentration- and time-dependent apoptosis were induced in both PC-3 and DU-145 cells (Figure 2C).

**Figure 3:**
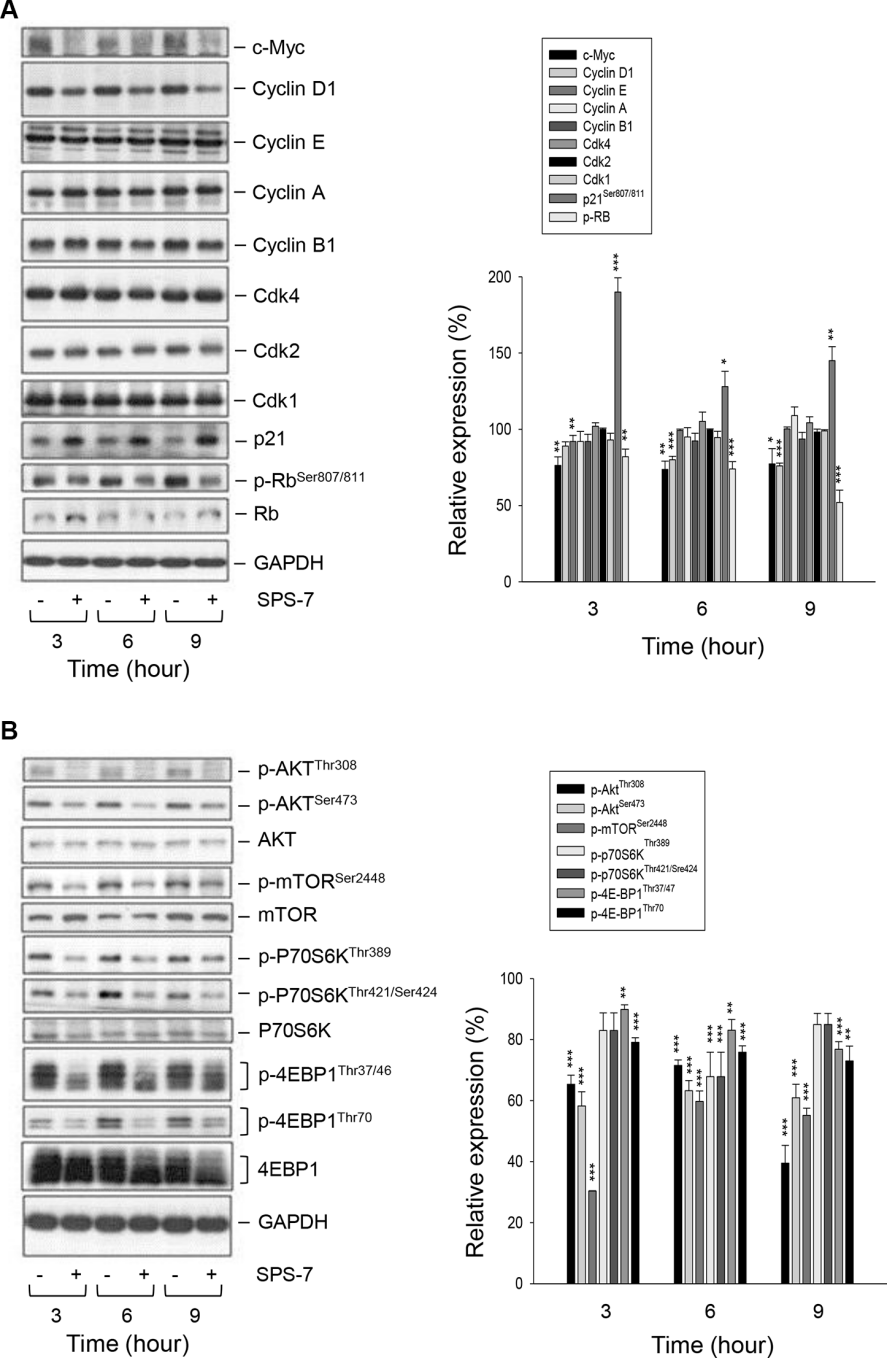
SPS-7 induces changes of expression levels of several proteins PC-3 cells were incubated in the absence or presence of SPS-7 (10 μM) for the indicated time. After the treatment, the cells were harvested and lysed for the detection of protein expressions of cell cycle regulators (**A**), and Akt/mTOR pathway signals (**B**) by Western blot analysis. The data are representative of three independent experiments. Data are expressed as mean ± SEM of three determinations. *P < 0.05, **P < 0.01 and ***P < 0.001 compared with the control of 100%.

Cell cycle progression is regulated by periodic activation of several Cdk/cyclin complexes. Cyclin D1 interacts with Cdk4 forming a complex that prompts the cells entering G1 phase. SPS-7-induced decrease inthe protein expression of cyclin D1 but notthe other cyclins correlated to G1 arrest (Figure 3A). Rb, a tumor suppressor responsible to G1 checkpoint, impedes an entry into S phase of the cell cycle. Cyclin D1/Cdk4 complex inhibits Rb by appropriate phosphorylation andreduces its association with E2F transcription factor, leading tothe activation of downstream gene transcription [23]. p21, the endogenous Cdk inhibitor, binds and inhibits the activities of cyclin D1/Cdk4 complexes and blocks the transition from G1 into S phase [24, 25]. As a consequence, SPS-7 decreased the level of Rb phosphorylation and dramatically increased p21 protein expression. The data agreed with the effects on G1 arrest and decreased level of cyclin D1 (Figure 3A). Notably, SPS-7 decreased the expression of c-Myc, an oncoprotein discovered to participate in many cellular functions including cell proliferation, apoptosis and transformation [26, 27]. c-Myc may collaborate with several growth factors and kinases in regulating cyclin D1 expression. Therefore, it is suggested that targeting c-Myc and cyclin D1 may be a good anticancer strategy [26, 28]. However, the role of c-Myc on cyclin D1 expression needs further clarification (please see below).

### SPS-7 inhibits the activation of Akt/mTOR pathway

A variety of reports have suggested the importance of the PI3K/Akt/mTOR pathway through p70S6K- and 4EBP1-mediated translational regulation on cell proliferation and survival [3–5, 29]. The data demonstrated the activities on the Akt/mTOR signaling including the phosphorylation of Akt at both Thr308 and Ser473 residues in the activation loop and hydrophobic motif of the kinase, respectively, and phosphorylation of mTOR at Ser2448 that was dependent on the kinase activity (Figure 3B) [30, 31]. Furthermore, phosphorylation of p70S6K was detected at both Thr421/Ser424 and Thr389 that were required in cooperation to achieve full kinase activation [32]. Phosphorylation of the translation repressor, 4EBP1, was also apparent at both Thr37/46 and Thr70, which were necessary for removing this repressor protein (Figure 3B) [33]. Consequently, SPS-7 showed a promising inhibition on these signaling pathways as evidenced by the inhibition of phosphorylation on the mentioned kinases and proteins (Figure 3B). Furthermore, SPS-7 induced the cleavage of caspases such as caspase-9, caspase-7 and PARP-1 (a substrate of caspase-3 and -7), indicating the caspase-dependent apoptosis (Supplementary Figure S1).

### c-Myc and Akt signals interactively regulate downstream pathways

**Figure 4:**
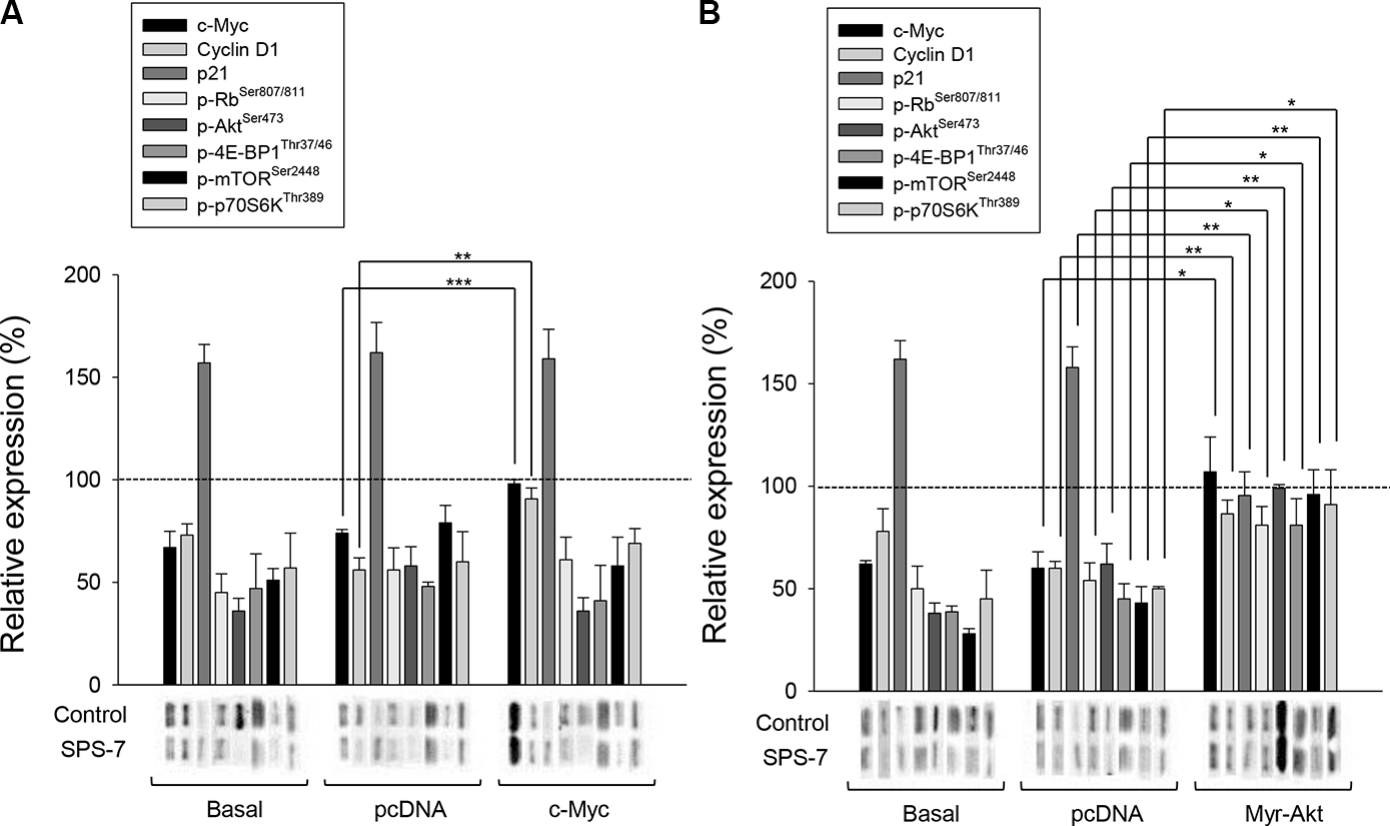
c-Myc and Akt involve in SPS-7-induced signaling pathways PC-3 cells were transfected with c-myc plasmid (**A**) or Myr-Akt plasmid (**B**). Then, the cells were incubated in the absence or presence of SPS-7 (10 μM) for nine hours. The cells were harvested and lysed for the detection of the indicated protein by Western blot analysis. The expression was quantified using the computerized image analysis system Image Quant (Amersham Biosciences). The data are expressed as mean ± SEM of three to five independent experiments. *P < 0.05, **P < 0.01 and ***P < 0.001.

c-Myc may collaborate with several receptors of growth factors, Ras/Raf mitogen-activated protein kinases cascade and PI3K/Akt through coordination in regulating cyclin D1 expression [26, 28, 34]. To determine the functional roles of c-Myc and Akt, plasmid transfection was performed and the effects on protein levels were examined using Western blot analysis. The data demonstrated that the cells transfected with c-myc expressed high levels of c-Myc protein, leading to the replenishment of cyclin D1 protein but not the other protein phosphorylation under the exposure to SPS-7 (Figure 4A). The data were verified by the knockdown of c-myc that led to the down-regulation of cyclin D1 protein expression (data not shown). The data revealed that c-Myc served as an upstream regulator on cyclin D1 dynamics. In contrast, overexpression of constitutively active Myr-Akt in the cells counteracted SPS-7-induced effects, including the induction of p21 protein expression and inhibitory effects of both c-Myc and cyclin D1 protein levels and the phosphorylation of Rb and mTOR pathway related proteins (Figure 4B). The parallel experiments also demonstrated that MK2206, an Akt inhibitor, exhibited similar effects to SPS-7 (data not shown). The data suggest that Akt functions as a key regulator on c-Myc expression and mTOR translational pathway.

### SPS-7 induces cytoprotective autophagy counteracting cell apoptosis

**Figure 5:**
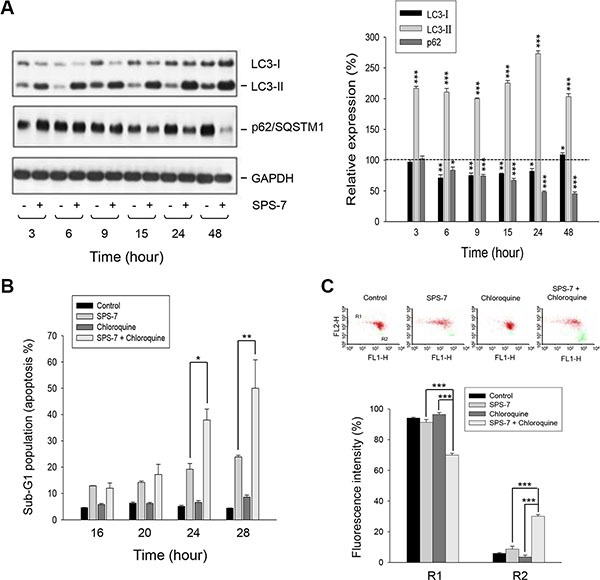
SPS-7 induces autophagy which counteracts apoptotic cell death PC-3 cells were incubated in the absence or presence of (**A**) 10 μM SPS-7 or (**B**, **C**) 10 μM SPS-7 and/or 10 μM chloroquine for the indicated time (A, B) or 24 hours (C). Cells were harvested and lysed for the detection of the indicated protein expression by Western blot analysis (A), or the cells were harvested for the detection of hypodiploid DNA content (apoptotic sub-G1 population) (B) or mitochondrial membrane potential (C) by flow cytometric analysis. Protein expression was quantified using computerized image analysis system ImageQuant (Amersham Biosciences, NJ, USA). Data are expressed as mean ± SEM of three to five independent experiments. *P < 0.05, **P < 0.01 and ***P < 0.001 compared with the control of 100%.

Autophagy, a ubiquitous self-cannibalization process on cellular organelles or proteins degraded and recycled through autophagosomes and lysosomes, may show cytoprotective effects to help maintain cell homeostasis or demonstrate another way to die [35, 36]. The activities of Akt and mTOR kinases have been suggested to be mechanistically linked to autophagy inhibition and tumorigenesis [37]. SPS-7 that displayed an inhibitory activity on these kinase pathways might represent a fundamental mechanism underlying the regulation of autophagy. The data demonstrated that SPS-7 facilitated the conversion of LC3-I to LC3-II (a hallmark of autophagosome formation) and the degradation of p62/SQSTM1 (an adaptor protein required for recognizing/loading cargo into autophagosomes) (Figure 5A), indicating autophagy induction. Chloroquine, a lysosomotropic agent for the inhibition of autophagy, by itself had little effect on PC-3 cells but dramatically enhanced SPS-7-induced apoptosis and the loss of mitochondrial membrane potential (Figure 5B and 5C). The data suggest that the autophagy may drive a cytoprotective mechanism counteracting apoptotic stress.

### SPS-7 has negligible effect on S1PR and lymphocyte homing

The S1PRs regulate a wide variety of biological processes including cell proliferation, migration, cytoskeleton organization, angiogenesis, endothelial cell chemotaxis, immune cell trafficking, mitogenesis and heart rate [38]. S1PRs areinvolved in immunemodulation and the suppression of innate immune responses from T cells. FTY720 is an S1PR modulators and immunosuppressor for the treatment of multiple sclerosis. Notably, recent studies show that FTY720 also induces anticancer activity in several cancer models through S1PR-independent signaling pathways that are distinct from the well-known immunosuppressive activity [39, 40]. Therefore, the development of anticancer agents without S1PR effect can be free from immunosuppression.

**Figure 6:**
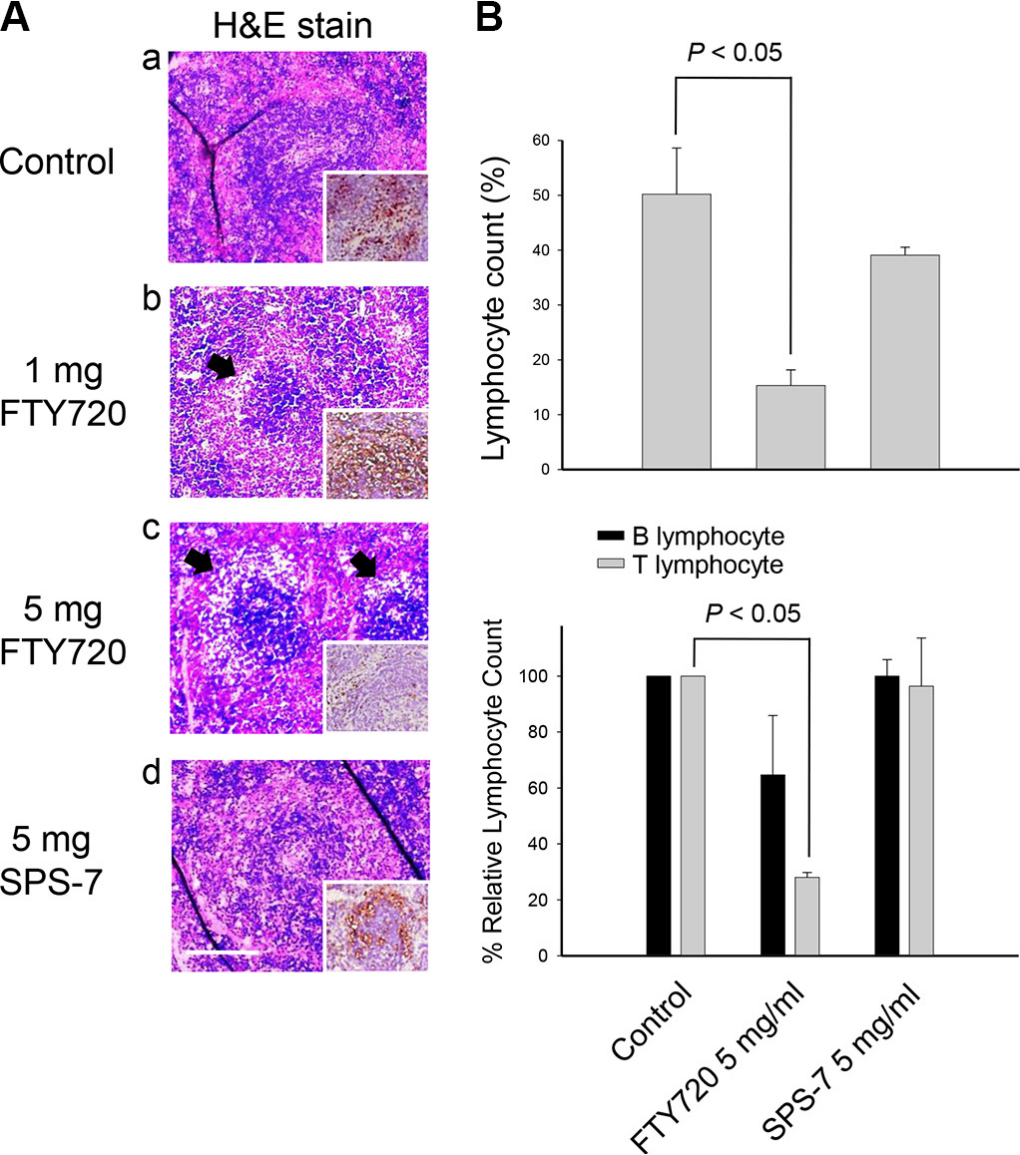
FTY720 but not SPS-7 induces immunosuppressive effect CD2F1 mice were treated with or without intraperitoneal FTY720 or SPS-7 for 6 hours. Whole blood was collected and the spleens were dissected. (**A**) Splenic sections were fixed, embedded with paraffin and de-paraffinized for H&E staining. IHC staining was used for detecting S1PR expression and performed by using UltraVision^™^ Quanto Detection System HRP DAB (Thermo Fisher Scientific, Waltham, MA, USA) (A, figure inset). (**B**) Blood lymphocyte count was performed using specific CD marker antibody conjugation and removal of erythrocytesas described in the Materials and Methods section. Then, lymphocyte count was determined by flow cytometric analysis (Becton Dickinson, Mountain View, CA). Data are expressed as mean ± SEM of five independent experiments. *P < 0.05 compared with the control.

The spleen tissues of immunocompetent CD2F1 hybrid mice were obtained for H&E staining and S1PR detection to address the effect on immune response. The spleen containsnon-lymphoid red pulp and lymphoid white pulp regions, mainly regulating erythrocyte disposal and antigens detect, respectively. Marginal zone, the region at the interface between red pulp and white pulp, traps particulate antigen from circulation and present to the lymphocytes of the spleen. Marginal zone B cells constitute a second major B cell population in the spleen. As a consequence, FTY720 at a six-hour treatment but not SPS-7 led to a profound depletion of marginal zone B cells (Figure 6A). Furthermore, FTY720 other than SPS-7 induced the down-regulation of S1P receptors (Figure 6A). The detection of circulating mature lymphocytes also showed that FTY720 but not SPS-7 induced a significant decrease in peripheral blood lymphocytes; more specifically, FTY720 significantly decreased T lymphocytes (Figure 6B). All together, it is indicative that FTY720 is an immunosuppressive agent. In contrast, SPS-7 at least at a short-term treatment shows little effect on modulating immune response.

### SPS-7 displays *in vivo* antitumor efficacy

**Figure 7:**
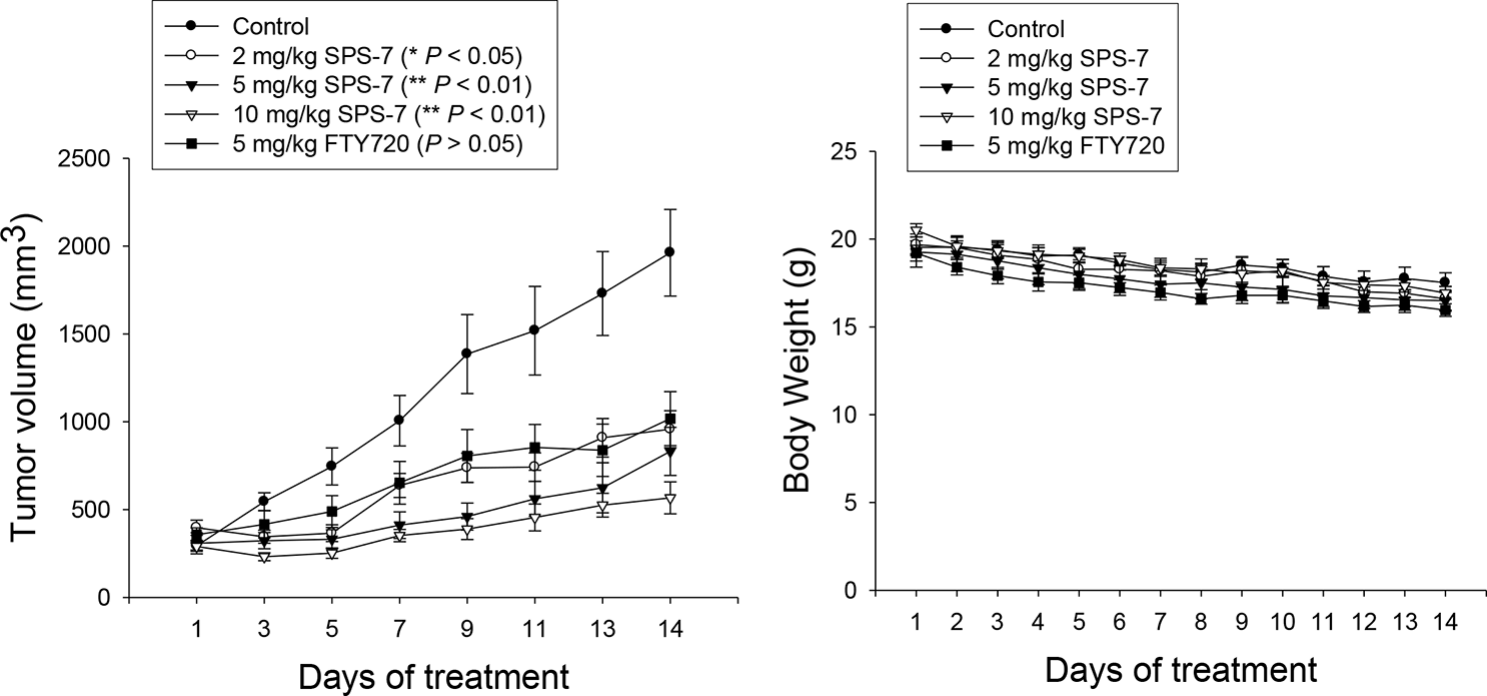
SPS-7 induces *in vivo* anti-tumor effect The nude mice were subcutaneously injected with PC-3 cells (10^7^ cell/mouse). The tumors were measured every two days. When the tumors had reached a volume of 200 to 300 mm^3^, the mice were divided into five groups (*n* = 6), and vehicle, SPS-7 or FTY720 was given intraperitoneally every day. Data are expressed as mean ± SEM of tumor size at indicated times post treatment with the compound. Weight change during treatment was also measured.

We subsequently carried out an *in vivo* study. The PC-3-derived cancer xenografts in nude mice were used as an *in vivo* model. As demonstrated in Figure 7, intraperitoneal (IP) administration of SPS-7 or FTY-720 caused a profound inhibition of tumor growth without a significant loss of body weight. The data also showed that the *in vivo* efficacy of SPS-7 was better than that of FTY720 (Figure 7).

## DISCUSSION

The U.S. Food and Drug Administration (FDA) have approved FTY720 as a firstline treatment in relapsing forms of multiple sclerosis. Recently, FTY720 has been suggested to demonstrate anticancer properties in a variety of cancer cells through multiple pathways [11–18, 41, 42]. Of note, some of these pathways are unrelated to FTY720's phosphorylation and immunosuppressive effects. In recent decades, clinical substantiation has been obtained for the anticipation that anticancer treatments based on developing the host's own defense mechanism can be beneficial [43]. To this end, keeping anticancer properties but diminishing the immunosuppressive activities is highly anticipated in developing FTY720 analogues. Owing to a phenylene moiety in FTY720's side chain conferring potent immunosuppressive activity, compound structure modification by displacing the phenylene ring with other ring moiety has generated several series of derivatives with reduced immunosuppressive effects while improving anticancer activities. SPS-7 was generated by displacing the phenylene ring with a triazole and a side chain with an optimized length. SPS-7 exhibited higher anti-proliferative and apoptotic activities than FTY-720 in PC-3, DU-145 and LNCaP cells.

Lymphocyte circulation is dependent on cell surface expression of S1P receptor. Down-regulation of S1P receptors in lymphocytes inhibits their exit from thymus and lymph nodes [44]. Splenic marginal zone B cells constitute a distinct naive B lymphoid lineage as a first line of defense against blood-borne pathogens [45]. They react to both thymus-dependent and -independent antigens, transport antigens to follicles to activate naive T cells and quickly differentiate into plasma cells, and initiate a rapid antibody response [45–48]. Depletion of these B cells can largely impair the related immune response and can be an indicator of immunosuppression. The data demonstrated that FTY720, but not SPS-7, induced the down-regulation of S1P receptors, the depletion of marginal zone B cells and a significant decrease in peripheral blood lymphocytes, indicating that FTY720 other than SPS-7 is an immunosuppressant.

The regulation of cell cycle machinery and checkpoint signaling pathways has provided a variety of targets for novel antineoplastic agents [49]. mTOR regulates various components involved in protein synthesis, in particular at G1 phase, including initiation and elongation factors, and biogenesis of ribosomes themselves [29–33]. The data revealed that SPS-7 caused a profound inhibition of mTOR and related translational pathways, resulting in the down-regulation of cyclin D1 protein expression and G1 arrest of the cell cycle. Recent studies have documented the functional link between c-Myc and mTOR in regulating protein synthesis, suggesting that mTOR-dependent phosphorylation of 4E-BP1 is necessary to cancer cell survival in c-Myc-dependent tumor initiation and maintenance [50]. Furthermore, in responding to oncogenic stimulation c-Myc may collaborate with PI3K/Akt in regulating cyclin D1 expression [26, 28, 34]. Because coordination of c-Myc and PI3K/Akt not only accelerates tumor formation but also renders its progression to a more aggressive phenotype, targeting these molecules is considered as good strategy for cancer therapy. Overexpression of c-Myc or Myr-Akt (a constitutively active mutant of Akt) using plasmid-based gene transfer provided evidence showing that the inhibition of c-Myc expression was responsible for the down-regulation of cyclin D1 protein levels to SPS-7 action. Moreover, our discovery that Myr-Akt overexpression completely abolished SPS-7-induced effects suggested that Akt functioned upstream of c-Myc and mTOR translational pathways.

PI3K/Akt is an early signaling molecule induced by growth factor receptor stimulation necessary for cell cycle entry and proliferation. PI3K/Akt activation occurs at immediate growth factor supplement and late G1 phase of the cell cycle. Similar to our work, multiple lines of evidence demonstrate that inhibition of late G1 PI3K/Akt activity leads to a decrease of c-Myc expression levels because of c-Myc destabilization [51]. Several signaling molecules through PI3K/Akt have been suggested to be involved in stabilizing c-Myc, including glycogen synthase kinase 3β, FoxO protein, NF-κB, signal transducer and activator of transcription 3 (STAT3) and mTOR [52, 53]. mTOR exists as two structurally distinct complexes, including the Akt substrate mTORC1 which regulating p70S6K and 4E-BP1/eIF4E, and mTORC2 which acting upstream of Akt [30, 31]. Our study revealed that the anti-proliferative and apoptotic effects of SPS-7 correlated with an inhibition of p70S6K and 4E-BP1 function, indicating that inhibition of cap-dependent protein synthesis directed the anticancer effect. Furthermore, c-Myc is short-lived with a half-life of 15 to 30 min, requiring persistent synthesis for keeping protein levels such that the blockade of cap-dependent translation would rapidly decrease c-Myc protein levels. In support of these findings, it has been reported that certain dual targeted PI3K/mTOR inhibitors efficiently killed primary c-Myc-driven B-cell lymphomas and human cell lines bearing IG-c-Myc translocations [54]. Altogether, our mechanistic interpretation offers a potential therapeutic opportunity for the treatment of HRPC with the inhibitor of PI3K/Akt/mTOR/c-Myc signaling pathways.

Autophagy and apoptosis are two key cellular processes that regulate cell fate with complicated and interconnecting signaling crosstalk. It has been widely reported that they can regulate each other through signaling pathways that control both processes [35–37]. For example, p53 can simultaneously induce autophagy through increased expression of the effector DRAM (damage-regulated autophagy modulator), a p53 target gene encoding a lysosomal protein that induces macroautophagy [55, 56]. Furthermore, PI3K/Akt activation is a way to inhibit apoptosis andtoblock autophagy [37, 56]. Several components of apoptosis and autophagy machinery can also regulate both processes, such as calpain, Fas-associated protein with death domain (FADD) and Bcl-2 family of proteins [56]. Our data showed that SPS-7 resulted in autophagic cellular program. Chloroquine, an inhibitor of autophagy, has been suggested to potentiate apoptotic cell death induced by various stimuli, indicating a cytoprotective role of autophagy. The data demonstrated that chloroquine significantly increased mitochondrial damage and apoptosis induced by SPS-7 suggesting that autophagy blunted SPS-7-induced anticancer effect.

**Figure 8:**
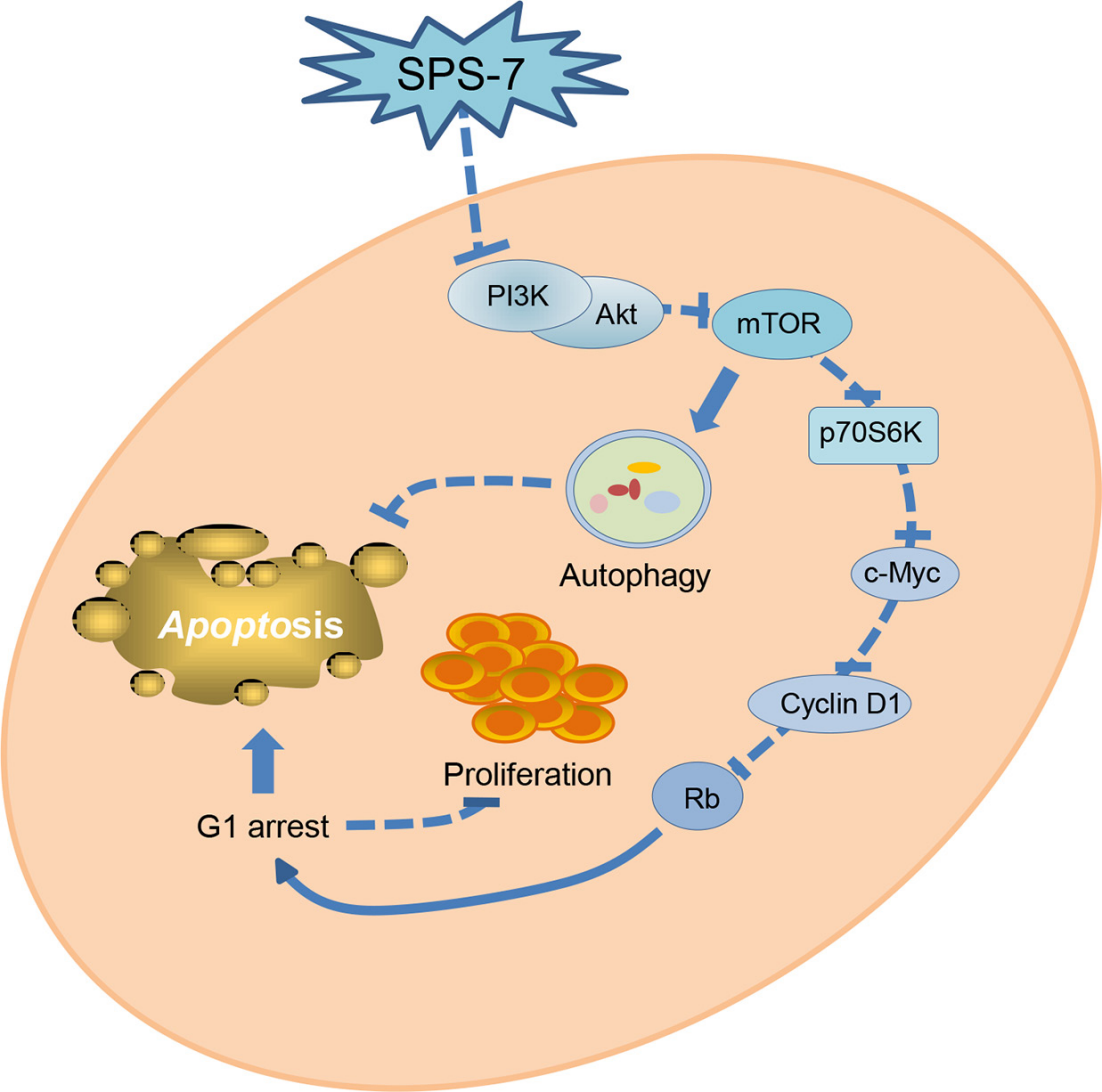
Schematic figure for SPS-7-mediated signaling pathways on the inhibition of cell proliferation and apoptosis induction SPS-7 inhibits the activation of PI3K/Akt, which *in turn* inhibits mTOR signaling pathway and activation of c-Myc-cyclin D1-Rb axis, leading to G1 arrest of the cell cycle and apoptosis. SPS-7 also induces the activation of cytoprotective autophagy that partly blunts the apoptosis. Therefore, combination of autophagy inhibitors has been suggested on developing anticancer agents targeting PI3K/Akt/mTOR pathways.

Taken together, the data suggest that SPS-7 is not an immunosuppressant while induces anti-proliferative and apoptotic signaling cascades against HRPC in a sequential manner (Figure 8). SPS-7 elicits an inhibitory effect on PI3K/Akt activity, leading to the inhibition of mTOR signaling pathways and c-Myc down-regulation which *in turn* decrease cyclin D1 protein expression and induce G1 arrest of the cell cycle and apoptosis. However, SPS-7 also activates cytoprotective autophagy that blunts apoptosis. Combination of autophagy inhibitors may potentiate apoptosis induced by agents targeting PI3K/Akt/mTOR/c-Myc axis and provide novel strategy for the development of SPS-7.

## MATERIALS AND METHODS

### Materials

RPMI 1640 medium, antibodies to CD3e, Pen-Strep-Ampho Sol (10,000 U/ml of Penicillin, 10 mg/ml of Streptomycin, 0.025 mg/ml of Amphotericin B) and fetal bovine serum (FBS) were obtained from GIBCO/BRL Life Technologies (Grand Island, NY). Antibodies to caspase-7 and CD45RA, and BD Pharm Lyse™ lysing solution were from BD Transduction Laboratories (BD Bioscience, San Jose, CA). Flow cytometry staining buffer was from eBioscience (San Diego, CA). Antibodies to cyclin D1, cyclin E, cyclin A, cyclin B1, cyclin-dependent kinase (Cdk) 4, Cdk2, Cdk1, *Poly* (ADP-ribose) polymerase (PARP), p21, CDC25A, α-tubulin, Bcl-2, Bcl-xL, Mcl-1, Bak, Bid, Bax, Bad, LC-3, c-myc (N262), c-myc siRNA and HRP-conjugated anti-mouse and anti-rabbit IgGs were obtained from Santa Cruz Biotechnology, Inc. (Santa Cruz, CA). Antibody to S1PR was from GENETEX (Irvine,CA). Antibodies to p-RB^Ser801/811^, caspase-9, caspase-7, p-Akt^Ser473^, p-Akt^Thr308^, p-p70s6K^Thr421/Ser424^, p-p70s6K^Thr389^, p-4E-BP1^Thr37/46^, p-4E-BP1^Thr70^ and GAPDH were from Cell Signaling Technologies (Boston, MA). NaHCO_3_, dithiothreitol, phenylmethylsulfonylfluoride (PMSF), sulforhodamine B (SRB), propidium iodide (PI), trichloroacetic acid (TCA), thymine deoxyriboside, leupeptin, NaF, NaVO_4_, chloroquine, Hank's balanced salt solution (HBSS) and all other chemical compounds were obtained from Sigma-Aldrich (St. Louis, MO). Bio-Red protein assay kit was from Bio-Red (Hercules, CA). Carboxyfluorescein succinimidyl ester (CFSE) was from Molecular Probes Inc. (Eugene, OR, USA). SPS compounds were synthesized and provided by our colleague (Dr. Duen-Ren Hou). The purity is more than 95% by the examination using HPLC and NMR.

### Tissue explants and cell culture

All human tissue samples were obtained following informed consent of the donors and after fullreview by the Ethics Review Committee at National Taiwan University Hospital. The prostate specimens were from males by transurethral resection of the prostate. All patients with prostatism histories were diagnosed to have benign prostate hyperplasia by rectal digital examination, transrectal sonography of prostate and urodynamic studies. Isolation of human prostatic cells from prostatic tissue explants was described in the previous study [57]. Prostate cancer cell lines were from American Type Culture Collection (Rockville, MD). Cells were cultured in RPMI 1640 medium with 10% FBS (*v/v*), penicillin (100 units/ml) and streptomycin (100 μg/ml). Cultures were maintained in a 37^°^C incubator with 5% CO_2_.

### SRB assays

Cells were seeded in 96-well plates in medium with 10% FBS. After 24 hours, cells were fixed with 10% TCA to represent cell population at the time of compound addition (TZ). After additional incubation of DMSO or the compound for 48 or 72 h, PC-3 or DU-145 cells were fixed with 10% TCA and SRB at 0.4% (w/v) in 1% acetic acid was added to stain cells. Unbound SRB was washed out by 1% acetic acid and SRB bound cells were solubilized with 10 mM Tris base. The absorbance was read at a wavelength of 515 nm. Using the following absorbance measurements, such as time zero (TZ), control growth (CTL), and cell growth in the presence of the compound (Tx), the percentage growth was calculated at each of the compound concentrations levels. Percentage growth inhibition was calculated as: [1-(Tx-TZ)/(CTL-TZ)] × 100%. Growth inhibition of 50% (IC_50_) is determined at the compound concentration which results in 50% reduction of total protein increase in control cells during the compound incubation.

### Lymphocyte preparation and flow cytometry

CD2F1 mice weighing 19 to 21 g were purchased from Lasco (BioLASCO Taiwan Co., Ltd). The mice were receiving intraperitoneal injection with FTY720, SPS-7 at 1 or 5 mg/kg, or vehicle. After 6 hours, whole blood was collected from the heart of the mice into microtainer tube and lymph lymphocytes were harvested from the spleen. Mice spleenswere dissected on cold HBSS buffer with 10% FBS and scraped/smashed with a sterile tip on Falcon^®^ 100 μm cell strainers into homogenized suspension. All samples were stained with PE conjugated rat anti-mouse CD45RA (Clone 14.8) and FITC conjugated hamster anti-mouse CD3e (clone 145-2C11) molecular complex for 30 minutes in darkness. Then, all samples were prepared after removal of erythrocytes using BD Pharm Lyse^™^ lysing solution (BD Bioscience, San Jose, CA) for 15 to 60 minutes at room temperature in the dark. Finally, all samples were washed 1X flow cytometry staining buffer and determined by flow cytometric analysis (Becton Dickinson, Mountain View, CA).

### Hematoxylin and eosin (H&E) and immunocytochemistry (IHC) staining

After the treatment, mice splenic sections were fixed with 10% formalin for 24 hours and embedded with paraffin. Tissue sections (7 μm) were de-paraffinized with xylene and washed with PBS. H&E staining was used for detecting marginal zone B cells. IHC staining was used for detecting S1PR expression and performed by using UltraVision^™^ Quanto Detection System HRP DAB (Thermo Fisher Scientific, Waltham, MA, USA). In brief, the sectionswere treated with Hydrogen Peroxide Block for 10 minutes and then Ultra V Block for 5 minutes. The sections were incubated with anti-rabbit S1PR antibody (1:200 dilutions) at 4°C for 1 hour and washed with cold PBS for 5 minutes twice. Then, thesections were incubated with primary Antibody Amplifier Quanto, HRP Polymer Quanto, DAB QuantoChromogen and DAB Quanto Substrate, and were determined by chromogenic detection according to the manufacturer's instructions.

### Cell proliferation assay with CFSE labeling

The cells were adjusted to a density of 10^6^ cells/ml and were treated with CFSE at a final concentration of 10 μM. After incubation at 37^°^C for 10 minutes, labeling was blocked by the addition of RPMI medium with 10% FBS and then, ice-cold treated for 5 minutes. After centrifugation, the cells were seeded in RPMI medium with 10% FBS for 24, 48 and 72 hours at 37^°^C under 5% CO_2_/95% air. After the treatment, the fluorescence intensity was determined by flow cytometric analysis (Becton Dickinson, Mountain View, CA). The cell proliferation was to be followed by monitoring decrease in label intensity in successive daughter cell generations [58]. The proliferation index and the cell populations of parent or different generations were calculated by Modfit LT Version 3.2 and WinList Version 5.0 software.

### Cell cycle synchronization

Synchronization of thecells was performed by thymidine block. Briefly, cells were treated with 2 mM thymidine in medium/10% FBS for 24 hours. After washing cells with PBS, the block was released by the incubation of cells in fresh medium/10% FBS (indicated as time zero), and cells were harvested at the indicated times. The cell-cycle progression was detected by flow cytometric analysis.

### Flow cytometric analysis of PI staining

After treatment, cells were harvested by trypsinization, fixed with 70% (*v/v*) alcohol at 4^°^C for 30 minutes and washed with PBS. The cells were centrifuged and resuspended with 0.5 ml PI solution containing Triton X-100 (0.1%, *v/v*), RNase (100 μg/ml) and PI (80 μg/ml). DNA content was analyzed with the flow cytometric analysis (Becton Dickinson, Mountain View, CA).

### Western blotting

After the treatment, the cells were harvested with trypsinization, centrifuged and lysed in 0.1 ml of lysis buffer containing 10 mM Tris-HCl (pH 7.4), 150 mM NaCl, 1 mM EGTA, 1% Triton X-100, 1 mM PMSF, 10 μg/ml leupeptin, 10 μg/ml aprotinin, 50 mM NaF and 100 mM sodium orthovanadate. Total protein was quantified, mixed with sample buffer and boiled at 90^°^C for 5 minutes. Equal amount of protein (30 μg) was separated by electrophoresis in SDS-PAGE, transferred to PVDF membranes and detected with specific antibodies. The immunoreactive proteins after incubation with appropriately labeled secondary antibody were detected with an enhanced chemiluminescence detection kit (*GE Healthcare* Life Sciences, Buckinghamshire, UK).

### Measurement of mitochondrial membrane potential (ΔΨ_m_)

JC-1, a mitochondrial dye staining mitochondria in living cells in a membrane potential-dependent fashion, was used to determine ΔΨ_m_. Cells were treated with or without the compound. Thirty minutes before the termination of incubation, the cells were incubated with JC-1 (final concentration of 2 μM) at 37°C for 30 minutes. The cells were finally harvested and the accumulation of JC-1 was determined using flow cytometric analysis (Becton Dickinson, Mountain View, CA).

### Transient transfection

The plasmids encoding c-Myc and Myr-Akt were used. For transfection, PC-3 cells were seeded into 60-mm tissue culture dishes with 30% confluence and grown for 24 hours to about 50% confluence. Each dish was washed with serum-free Opti-MEM (Life Technologies, Grand Island, NY), and 2 ml of the same medium was added. Aliquots containing expression vector or a control plasmid in serum-free Opti-MEM were transfected into cells using Lipofectamine 2000 (Invitrogen, Carlsbad, CA) following the manufacturer's instructions. After the incubation for 6 hours at 37°C, cells were washed with medium and incubated in 10% FBS-containing RPMI-1640 medium for 48 hours. Then, the cells were treated with or without SPS-7. The Western blot analysis was performed.

### *In Vivo* antitumor models

PC-3-derived cancer xenografts in nude mice were used as an *in vivo* model. The nude mice were subcutaneously injected with PC-3 cells (10^7^ cell/mouse). The tumors were measured every 2 days. When the tumors had reached a volume of 200 to 300 mm^3^, the mice were divided into five groups (*n* = 6) and the compound treatment was initiated. The compound was dissolved in 2% Tween 80. Vehicle (2% Tween 80) or the compound was given intraperitoneally every day. The length (l) and width (w) of the tumor were measured every 2 days. The tumor volume was calculated as lw^2^/2. Animals were euthanized when a body weight loss of 15% at any time point or the tumor size reached 2000 mm^3^. The protocols of the *in vivo* study were approved by the Animal Care and Use Committee at National Taiwan University.

### Data analysis

Data are presented as the mean ± SEM for the indicated number of separate experiments. Statistical analysis of data for multiple groups is performed with one-way analysis of variance followed by Dunnett's multiple comparisons test. Single comparisons of appropriate groups were done with Student's *t*-test. *P*-values less than 0.05 are statistically considered significant.

## SUPPLEMENTARY MATERIALS METHODS AND FIGURE


