# Supplementary material for: Non-immunosuppressive triazole-based small molecule induces anticancer activity against human hormone-refractory prostate cancers: the role in inhibition of PI3K/AKT/mTOR and c-Myc signaling pathways
Source: Oncotarget. 2016 Oct 19;7(47):76995–7009. doi: 10.18632/oncotarget.12765 (PMC5363565; doi:10.18632/oncotarget.12765)
Supplement: Supplementary file 1 [file oncotarget-07-76995-s001.pdf]

Non-immunosuppressive triazole-based small molecule induces anticancer activity against human hormone-refractory prostate cancers: the role in inhibition of PI3K/AKT/mTOR and c-Myc signaling pathways

Supplementary Materials

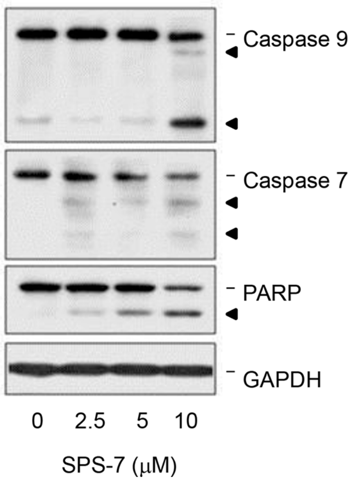

Supplementary Figure S1: : Effect of SPS-7 on the expression of caspases and PARP-1. PC-3 cells were incubated in the absence or presence of SPS-7 for 24 h. After the treatment, the cells were harvested and lysed for the detection of protein expressions by Western blot analysis. The data are representative of three independent experiments.

Scheme I. Synthesis of SPS-7

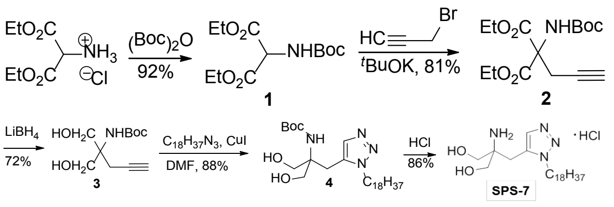

Diethyl 2-((*tert*-butoxycarbonyl)amino) malonate (1)

To a solution of diethylaminomalonate hydrochloride (211.9 1.01 mmol, 1.0 equiv) and triethylamine (204.5 mg, 2.02 mmol, 2.0equiv) indichloromethane (5.0 mL) was added di-*tert*-butyl dicarbonate (242.4 mg, 1.11 mmol, 1.1equiv). The reaction was stirred at rt for 12 h, then quenched with water, extracted with ethyl acetate, and excess solvent was evaporated under vacuum to give compound1(255.8 mg, 0.93mmol, 92%) as a colorless oil. ^{1}H NMR (300 MHz, $CDCl_3$). 5.54 (d, *J* =8.0 Hz,1H), 4.93 (d, *J* =8.0 Hz,1H), 4.36-4.12 (m, 4H), 1.42 (s, 9H), 1.23 (t, *J* =7.0 Hz,6H);^{13}C NMR (75 MHz, $CDCl_3$)166.6, 154.7, 80.5, 62.4, 57.5, 28.1, 13.9; HRMS (ESI$^+$) calcd for[M+Na]$^+$($C_{12}H_{21}NO_6Na$), 298.1267, found298.1269.

Diethyl 2-(tert-butoxycarbonylamino)-2-(prop-2-ynyl)malonate(2)

To a solution of diethyl 2-(*tert*-butoxycarbonylamino) malonate (1, 278.1 mg, 1.01 mmol, 1.0 equiv) and propargyl bromide (144.1 mg, 1.21mmol, 1.2equiv) in1,4-dioxane (5.0 mL) was added potassium-*tert*-butoxide (147.9 mg, 1.21mmol, 1.2equiv). The reaction was stirred heated at 90°C for 18 h, then quenched with water, extracted with ethyl acetate, and excess solvent was evaporated under vacuum. The crude product was purified by column chromatography (SiO_2: EtOAc/*n*-hexane, 35:65; R_f 0.56) to give compound2 (256.3 mg, 0.81 mmol, 81%) as a colorless oil. ^{1}H NMR (300 MHz, $CDCl_3$)δ 6.09 (s, 1H), 4.26–4.22 (m, 4H), 3.22 (s, 2H), 1.96 (s, 1H), 1.41 (s, 9H), 1.24 (t, *J* = 7.0 Hz,6H);^{13}C NMR (75 MHz, $CDCl_3$)166.8, 153.9, 80.5, 78.4, 71.3, 65.5, 62.8, 28.1, 24.3, 13.9.

tert-Butyl 1-hydroxy-2-(hydroxymethyl)pent-4-yn-2-ylcarbamate(3)

To a solution of diethyl diethyl 2-(*tert*-butoxy-carbonylamino)-2-(prop-2-ynyl)malonate (2, 316.4 mg, 1.01 mmol, 1.0 equiv) indry THF (5.0 mL) was added lithium borohydride (87.6 mg, 4.04 mmol, 4.0 equiv), and dry methanol (1.0 mL). The reaction was stirred heated at 0°C for 6 h, then quenched with water, dried over anhydrous magnesium sulfate, and excess solvent was evaporated under vacuum. The crude product was purified by column chromatography (SiO_2: EtOAc/*n*-hexane, 50:50;R_f 0.60) to give compound3 (166.7 mg, 0.73 mmol, 72%) as a colorless oil.^{1}H NMR (300 MHz, $CDCl_3$)*d* 5.24 (s, 1H), 3.84 (d, *J* = 6.9 Hz,2H), 3.62 (d, *J* = 6.9 Hz,2H), 3.58 (s, 2H), 2.53 (d, *J* = 2.7 Hz,2H), 2.06 (t, *J* = 2.7 Hz,1H), 1.42 (s, 9H);^{13}C NMR (75 MHz, $CDCl_3$) 156.4, 80.5, 79.4, 71.6, 65.0, 58.5, 28.2, 22.9.

tert-Butyl 1,3-dihydroxy-2-((1-octadecyl-1H-1,2,3-triazol-4-yl)methyl)propan-2-ylcarbamate(4)

To a solution ofalkyne3(59.6 mg, 0.26 mmol)and 1-azidooctadecane (92.2 mg, 0.31mmol)inDMF (2.0 mL) was added CuI (5.7 mg, 0.03mmol. The reaction was stirred at rt for 16 h, then quenched with water, extracted with ethyl acetate, and excess solvent was evaporated under vacuum. The crude product was purified by column chromatography (SiO_2: EtOAc/*n*-hexane, 55:45; R_f 0.64) to give compound4 (120.7 mg, 0.23 mmol, 88%) as solid (m.p. 82–83°C).^{1}H NMR (300 MHz, $CDCl_3$)δ 7.47 (s, 1H), 5.59 (s, 1H), 4.77 (s, 2H), 4.27 (t,*J* =7.2 Hz,2H), 3.58 (d, *J* =10.8 Hz,2H), 3.46 (d, *J* =12.0 Hz,2H), 3.11 (s, 2H), 1.87-1.85 (m, 2H), 1.39 (s, 9H), 1.22 (s, 30H), 0.84 (t, *J* =6.6 Hz,3H);^{13}C NMR (75 MHz, $CDCl_3$) 156.9, 143.6, 123.4, 80.0, 65.1, 59.6, 50.4, 31.9, 30.2, 29.6, 29.5, 29.3, 28.9, 28.3, 27.7, 26.4, 22.6, 14.1; HRMS (ESI$^+$) calcd for [M+Na]$^+$($C_{29}H_{56}N_4O_4Na$), 547.4199, found547.4193.

1,3-Dihydroxy-2-((1-octadecyl-1H-1,2,3-triazol-4-yl)methyl)propan-2-aminium chloride (SPS-7)

A mixture of 4 (40.9mg, 0.078mmol), 10%methanolicHCl (0.28 mL, 0.78mmol), and methanol (1.0 mL) was stirred at rt for 6 h.The solvent of removed and the residue was washed with diethyl etherto give SPS-7 (30.8 mg, 0.067 mmol, 86%) as a white solid.^{1}H NMR (300 MHz, $(CD_3)_2SO$)δ 7.99 (s, 1H), 7.94 (s, 3H), 4.29 (t, *J* = 6.6 Hz,2H),3.43 (s, 4H),2.94 (s, 2H), 1.81-1.73 (m, 2H), 1.21 (s, 30H), 0.83(t, *J* = 6.2 Hz,3H);^{13}C NMR (75 MHz, $(CD_3)_2SO$)140.5, 124.8, 61.2, 60.6, 49.8,31.7, 30.1, 29.49, 29.48, 29.47, 29.46, 29.3, 29.1, 28.9, 27.2, 26.3, 14.1; HRMS (ESI$^-$) calcd for[M-H]$^-$($C_{24}H_{48}ClN_4O_2$) 459.3466, found459.3468.
